# Assessment of the circulating cell-free DNA marker association with diagnosis and prognostic prediction in patients with lymphoma: a single-center experience

**DOI:** 10.1007/s00277-017-3043-5

**Published:** 2017-06-16

**Authors:** Mao Li, Yongqian Jia, Juan Xu, Xiaomin Cheng, Caigang Xu

**Affiliations:** Hematology Research Laboratory, Department of Hematology, West China Hospital, Sichuan University, 37#, Guoxue Lane, Chengdu, Sichuan 610041 China

**Keywords:** Lymphoma, Circulating DNA, Integrity, Prognosis

## Abstract

Circulating cell-free DNA (ccfDNA) has been shown to be associated with the clinical characteristics and prognosis of cancer patients. Our objective was to assess whether the concentration and integrity index of ccfDNA in plasma may be useful for diagnosing and monitoring the progression of patients with lymphoma. We included plasma samples from 174 lymphoma patients and 80 healthy individuals. The total concentration of ccfDNA was determined using a fluorometry method, and the DNA integrity index (DII), which is the ratio of longer to shorter DNA fragments, for the APP gene was detected using real-time quantitative PCR. The median levels of the ccfDNA concentration and the DII in patients with lymphoma were significantly higher than those in controls (both *P* < 0.0001). Increases in the ccfDNA concentration and the DII were associated with advanced stage disease, elevated lactate dehydrogenase levels, and a higher prognosis score. In patients with diffuse large B cell lymphoma (DLBCL), high levels of ccfDNA (both concentration and the DII) showed an inferior 2-year progression-free survival (PFS) (*P* = 0.001; *P* < 0.0001, respectively). Our study provides quantitative and qualitative evidence in favor of using ccfDNA analysis in lymphoma patients for diagnostic and prognostic assessments.

## Introduction

Lymphoma, which is a common cancer of the hematopoietic system, represents a heterogeneous collection of diseases with different biological characteristics and clinical outcomes. In China, more than 80,000 new cases of lymphoma and an estimated 52,000 deaths attributed to lymphoma were reported in 2015 [1]. The overall survival of patients with lymphoma tends to be poor primarily because at the time of diagnosis, most patients are already in the late stages and thus lose the opportunity for timely treatment [2]. Therefore, there is an urgent need for a noninvasive tool to improve the diagnosis and prognostic evaluation of lymphoma patients.

Circulating cell-free DNA (ccfDNA) are DNA fragments circulating within the bloodstream and can be obtained by a simple blood collection. Mendel and Metais first reported the existence of ccfDNA in 1948 [3], and the presence of ccfDNA has been identified in inflammatory disease [4], autoimmune disease [5], trauma [6], and myocardial infarction [7]. Recently, quantifying ccfDNA levels has been proposed as a potential biomarker to detect malignant tumors. A study by Leon et al. [8] suggested that compared with that of healthy individuals, the concentration of ccfDNA was significantly increased in cancer patients; furthermore, this phenomenon was confirmed by other studies on breast tumors [9], colorectal cancer [10], lung cancer [11], and other cancer types [12]. The ccfDNA released into the bloodstream is thought to originate from either passive release from apoptotic and necrotic cells or active secretion from nucleated cells such as lymphocytes. Unlike uniformly truncated DNA released from apoptotic cells, ccfDNA released from necrotic tumor cells varies in size, which may lead to elevated levels of long fragments of DNA in the plasma of patients with malignant diseases [13]. Several studies have demonstrated that compared to that of healthy individuals, the DNA integrity index (DII), which is calculated as the ratio of longer to shorter DNA fragments, was increased in patients with cancers [14–16].

Although the presence of ccfDNA has been widely studied in malignant tumors, there are only few reports regarding ccfDNA in lymphoproliferative diseases. It has been hypothesized that ccfDNA may be a good candidate for diagnosis and prognosis of lymphoma because some studies have shown that the concentration of ccfDNA could be a diagnostic and predictive biomarker in lymphoma patients [17, 18]; however, to the best of our knowledge, there are no studies that have investigated the predictive value of ccfDNA integrity.

Here, we assessed the value of the concentration and integrity of ccfDNA in diagnosing lymphoma patients and analyzed these parameters to identify associations with clinical characteristics and prognosis.

## Materials and methods

### Population

There were 174 lymphoma patients enrolled in this study. Among the included lymphoma cases, 126 were B cell non-Hodgkin lymphoma (B-NHL), with diffuse large B cell lymphoma (DLBCL, *n* = 98) as the most common subtype. There were 18, 9, and 21 patients diagnosed with HL, T cell non-HL (T-NHL), and extranodal NK/T cell lymphoma (NK/TCL), respectively. The diagnosis of all patients was histologically confirmed. Patient samples were obtained from West China Hospital of Sichuan University between September 2014 and January 2016. Plasma samples were collected at the time of initial diagnosis. The median follow-up time was 13.5 months and ranged from 1 to 36 months. The main patient characteristics are provided in Table 1.

**Table 1:** Main characteristics of patients included in the study

	Total	HL	B-NHL		T-NHL	NK/TCL
			DLBCL	Others		
	(*n* = 174)	(*n* = 18)	(*n* = 98)	(*n* = 28)	(*n* = 9)	(*n* = 21)
Gender						
Male	107	10	61	17	6	13
Female	67	8	37	11	3	8
Age (range), years						
≤60	117	15	61	16	7	18
>60	57	3	37	12	2	3
Stage						
I–IIA	69	2	42	11	1	14
IIB–IV	105	16	56	17	8	7
B-symptoms						
No	91	6	60	14	2	9
Yes	83	12	38	14	7	12
Bulky disease						
No	153	13	84	26	9	21
Yes	21	5	14	2	0	0
Lactate dehydrogenase						
Normal	96	9	48	17	8	14
Elevated	78	9	50	11	1	7
IPI						
Low (0 or 1)	56	–	39	8	3	6
Intermediate low (2)	38	–	22	5	1	10
Intermediate high (3)	27	–	13	8	1	5
High (4 or 5)	35	–	24	7	4	0
IPS						
0–2	–	9	–	–	–	–
≥ 3	–	9	–	–	–	–

Data were presented as *n*

HL, Hodgkin’s lymphoma; B-NHL, B cell non-Hodgkin’s lymphoma; DLBCL, diffuse large B cell lymphoma; Others group included 9 follicular lymphoma, 7 small cell lymphoma/chronic lymphocytic leukemia, 6 marginal zone lymphoma, 5 mantle cell lymphoma and 1 Burkitt lymphoma; T-NHL, T cell non-Hodgkin’s lymphoma, included in 4 peripheral T cell lymphoma, 3 anaplastic large-cell lymphoma, 2 angioimmunoblastic T cell lymphoma; NK/TCL, extranodal NK/T cell lymphoma; IPI, international prognostic index; IPS, international prognostic score

A control group consisting of 80 healthy individuals was included to establish the normal range of ccfDNA levels in plasma. Informed consent was obtained from patients and controls according to the institutional guidelines, and the study was approved by the Human Research Ethics Committee of the West China Hospital of Sichuan University.

### Sample collection and DNA extraction

Peripheral blood samples were collected into tubes containing EDTA and centrifuged (2000×*g*, 10 min, 4 °C) within 4 h of collection. To prevent cellular DNA contamination, the plasma supernatants were carefully removed and recentrifuged (16,000×*g*; 10 min at 4 °C). The prepared plasma samples were stored at −80 °C until further analysis.

ccfDNA was extracted using the MagMAX™ Cell-Free DNA Isolation Kit (Applied Biosystems, Carlsbad, CA, USA) following the manufacturer’s instructions. The isolated ccfDNA was eluted in 20 μl of the provided solution and stored at −20 °C prior to quantitative and qualitative analyses.

### Quantification of circulating cell-free DNA

Quantification of ccfDNA was performed by fluorometry using the Qubit® dsDNA HS Assay Kit (Molecular Probes, Eugene, OR, USA) according to the manufacturer’s protocols, and the plates were read in a Qubit® 3.0 fluorometer (Invitrogen, Carlsbad, CA, USA). The ccfDNA concentrations were determined from a standard curve obtained using the standard stock (provided by the manufacturer). Two repeated tests were performed for each set of concentration measurements and the resulting data were averaged.

### Real-time quantitative PCR of DNA fragments

The integrity of ccfDNA in plasma was determined by quantitative real-time PCR. The quantitative assay was based on amplification of the APP gene (amyloid beta precursor protein, chromosome 21q21.3, accession NM_000484); the lengths of the two amplicons were 67 and 180 bp and were obtained using two primer pairs as reported previously [19]. The primer set for APP67 amplified both shorter (truncated by apoptosis) and longer DNA fragments (truncated by nonapoptotic means), whereas the APP180 amplified only the longer DNA fragments.

Quantification of ccfDNA fragments was performed using the CFX96 Touch™ Real-Time instrument (Bio-Rad, Hercules, CA, USA). The reaction mixture for each direct quantitative real-time PCR comprised 12.5 μl of SYBR® Premix Ex Taq™ II (TaKaRa, Shiga, Japan), 1 μl (0.4 μM) of each primer, and 2 μl DNA in a total reaction volume of 20 μl. The sequences of the primers are as follows: APP67: forward, 5′-TCAGGTTGACGCCGCTGT-3′ and reverse, 5′-TTCGTAGCCG TTCTGCTGC-3′; and APP180: forward, 5′-TCAGGTTGACGCCGCTGT-3′ and reverse, 5′-TCTATAAATGGACACCGATGGGTAGT-3′. The real-time PCR protocol included an initial denaturation step at 95 °C for 30 s followed by 40 cycles of denaturation at 95 °C for 5 s and annealing at 64 °C for 30 s. A melting curve analysis was conducted to confirm PCR product specificity, and the DNA copy number of each sample was determined using a standard curve with serial dilutions (2 × 10^−1^−2 × 10^5^ ng/ml) of human genomic DNA (Promega, Madison, WI, USA; 200 μg/ml). All samples were analyzed in duplicate, and a negative control (without template) was included in each plate. All quantitative real-time PCR assays were performed in a blinded fashion without knowledge of the specimen identity.

## Statistical analysis

Comparison of the relative expression levels of ccfDNA (including concentration and the DII) between patients and controls was performed by applying the nonparametric Mann-Whitney test. Analysis of receiver operating characteristics (ROC) curves and binary logistic regression were performed to evaluate the diagnostic performance of the ccfDNA concentration and the DII in lymphoma. The relationship between the levels of ccfDNA and the clinicopathological features of lymphoma patients was estimated as appropriate per variable type. In detail, differences in the ccfDNA levels among the different groups of categorical parameters were analyzed by either the nonparametric Mann-Whitney test for binary variables or the respective Kruskal-Wallis test for the parameters consisting of several independent groups. The Kaplan-Meier survival analysis approach and Cox proportional hazard regression analysis were used to determine progression-free survival (PFS). PFS was defined as the time from initial diagnosis until disease progression/relapse or death from any cause. A two-sided *P* < 0.05 indicated statistical significance. All analyses were performed using SPSS software version 22.0 (IBM Corp., Armonk, NY) and MedCalc software version 15.10 (MedCalc Software, Ostend, Belgium).

## Results

### Concentration of circulating cell-free DNA in healthy individuals and lymphoma patients

**Fig. 1:**
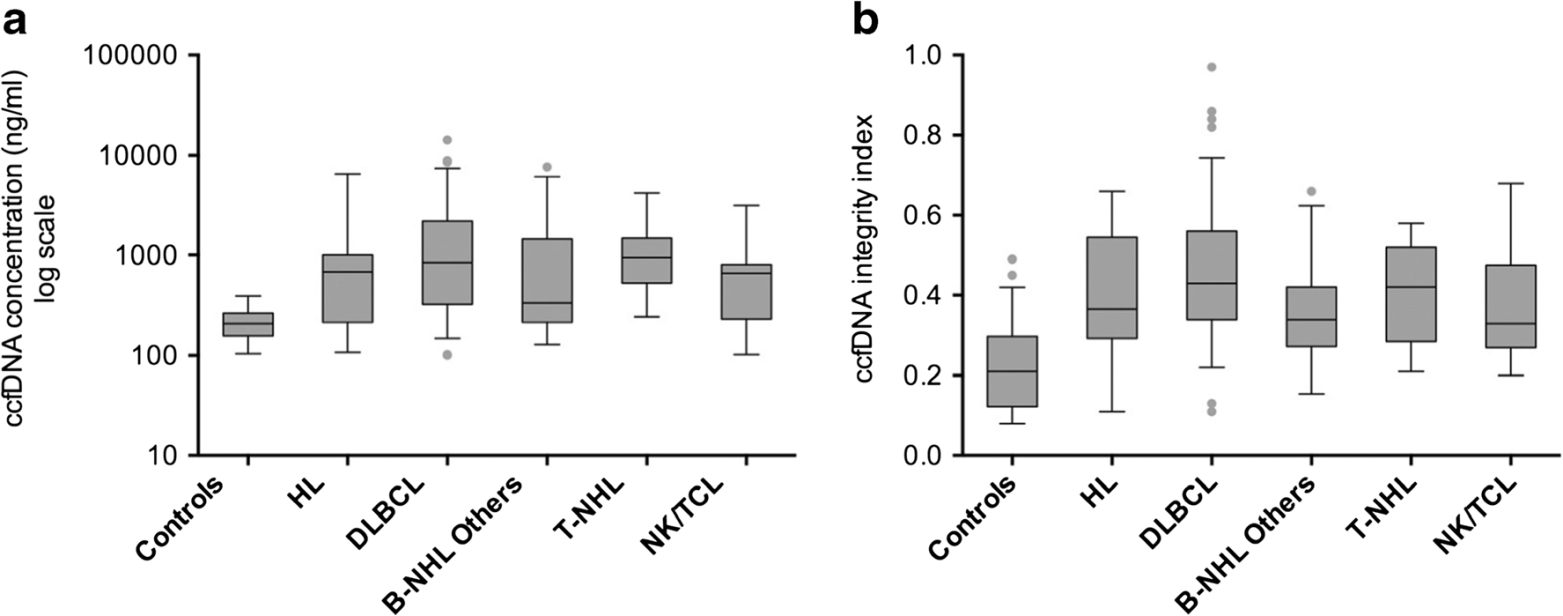
Circulating cell-free DNA levels in the plasma of lymphoma patients. Box plots showed circulating cell-free DNA levels of **a** concentrations and **b** DII in healthy controls and patients with lymphoma, presented as median value (*black line*), interquartile range (*box*), and 5th and 95th percentiles (*whiskers*)

The median concentration of ccfDNA in the 80 healthy individuals tested was 209.0 ng/ml (mean 222.5 ng/ml, range 100.0–456.0 ng/ml); however, in patients with lymphoma (*n* = 174), this concentration was significantly higher (median 686.0 ng/ml, *P* < 0.0001) with greater variability among the patients (mean 1407.6 ng/ml, range 100.0–14,180.0 ng/ml). Elevated ccfDNA concentration levels were observed in patients with HL (*n* = 18, median 681.0 ng/ml, mean 988.8 ng/ml, *P* = 0.001), DLBCL (*n* = 98, median 845.0 ng/ml, mean 1722.2 ng/ml, *P* < 0.0001), other types of B-NHL (*n* = 28, median 332.0 ng/ml, mean 1096.3 ng/ml, *P* < 0.0001), T-NHL (*n* = 9, median 942.0 ng/ml, mean 1247.0 ng/ml, *P* < 0.0001), and NK/TCL (*n* = 21, median 662.0 ng/ml, mean 782.7 ng/ml, *P* < 0.0001) (Fig. 1a).

To assess the diagnostic accuracy of the ccfDNA levels in lymphoma patients, an ROC curve analysis was conducted, and the area under the curve (AUC) was calculated as 0.75 (95% CI 0.66–0.84, *P* = 0.001) for HL, 0.86 (95% CI 0.80–0.90, *P* < 0.0001) for DLBCL, and 0.79 (95% CI 0.70–0.87, *P* < 0.0001) for NK/TCL, suggesting a moderate discriminatory power. For HL, the sensitivity and specificity were 61 and 100%, respectively, at a cut-off of 456.0 ng/ml; for DLBCL, 73 and 94% at a cut-off of 340.0 ng/ml, and for NK/TCL, 71 and 96% at a cut-off of 392.0 ng/ml.

Based on these results, elevated levels of ccfDNA were associated with a higher risk of lymphoma. When analyzed as a logistic regression model, a 10.0 ng/ml increase in the ccfDNA concentration increased the lymphoma risk by 7.3% (odds ratio 1.073; 95% CI 1.045–1.102).

### Integrity of circulating cell-free DNA in healthy individuals and lymphoma patients

The DII was calculated as the ratio of the quantitative real-time PCR results using the two primer sets as follows: Q_APP180_/Q_APP67_. Because the annealing sites of APP67 are contained within the APP180 annealing sites, the DII would be 1.0 when the template DNA was not truncated and 0.0 when all of the template DNA was truncated into fragments smaller than 180 bp.

The median DII in patients with lymphoma was 0.39 (mean 0.42, range 0.11–0.97), which was significantly higher than that of the normal control subjects (median 0.21, mean 0.22, range 0.07–0.49; *P* < 0.0001). Elevated DII values were observed in patients with HL (median 0.37, mean 0.40, *P* < 0.0001), DLBCL (median 0.43, mean 0.46, *P* < 0.0001), other types of B-NHL (median 0.34, mean 0.35, *P* = 0.002), T-NHL (median 0.42, mean 0.41, *P* = 0.008), and NK/TCL (median 0.33, mean 0.38, *P* = 0.001), respectively (Fig. 1b).

**Fig. 2:**
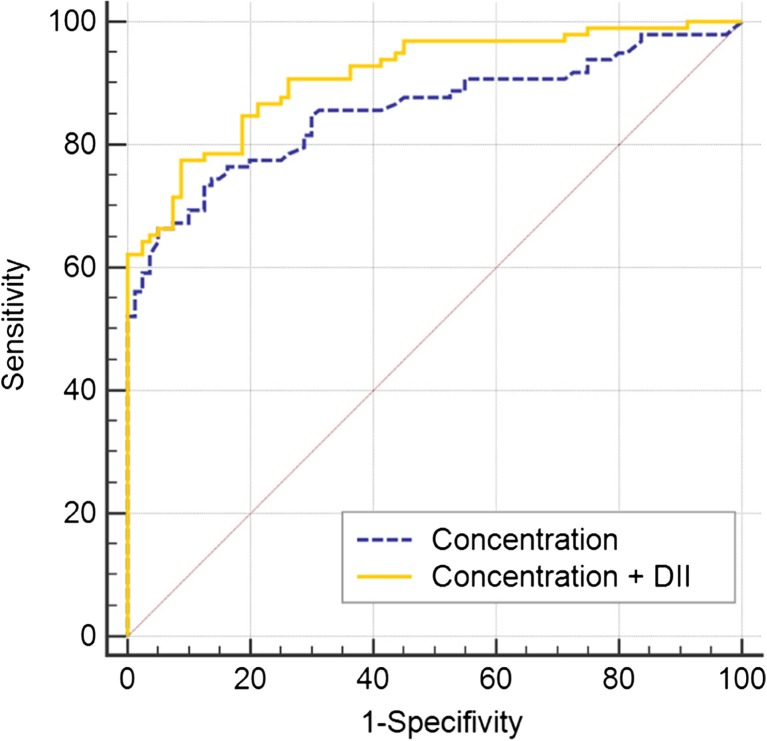
The receiver operating characteristic curves of the circulating cell-free DNA concentration and its combination with DII in patients with DLBCL. The AUC of concentration and model of concentration + DII was 0.86 (95% CI 0.80–0.90) and 0.91 (95% CI 0.86–0.95), respectively (*Z* = 2.697, *P* = 0.007). DII, DNA integrity index; AUC, area under the curve

Using the ROC analysis, the AUC of the ccfDNA DII was 0.83 (95% CI 0.74–0.90, *P* < 0.0001) for HL, 0.90 (95% CI 0.83–0.93, *P* < 0.0001) for DLBCL, and 0.81 (95% CI 0.72–0.88, *P* < 0.0001) for NK/TCL. Additionally, the ROC analysis assessed whether a combination of the raw ccfDNA concentration and the DII could improve the diagnostic ability compared with using the concentration alone. The results showed that the AUC was significantly increased after the addition of the DII to the ccfDNA concentration in patients with DLBCL (0.86 vs. 0.91; *Z* = 2.697, *P* = 0.007; Fig. 2); whereas in patients with either HL or NK/TCL, there was only a trend of increased AUC observed (0.75 vs. 0.84; *Z* = 1.714, *P* = 0.087 and 0.79 vs. 0.88; *Z* = 1.646, *P* = 0.0997, respectively).

### Correlation of circulating cell-free DNA and clinicopathological features in lymphoma patients

To assess the clinical significance of ccfDNA levels, we analyzed the clinicopathological correlations in the total lymphoma group but restricted the analysis to patients with DLBCL; in this patient subset, the adverse prognostic factors advanced stage disease (stage IIB–IV) and elevated LDH levels were associated with increased ccfDNA levels and DII (Table 2). In patients with DLBCL, the presence of B-symptoms was also correlated with increases in both the ccfDNA concentration and the DII. As a consequence, patients with an adverse prognostic score had higher levels of ccfDNA, which was significant among all lymphoma patients and DLBCL patients [20, 21]. Otherwise, no statistical significance was observed when the ccfDNA concentration and the DII were analyzed with regard to other clinical parameters such as age, gender, and bulky disease.

**Table 2:** Plasma circulating cell-free DNA levels and clinicopathologic features in lymphoma patients

	All patients		DLBCL	
	Concentration	DII	Concentration	DII
Gender				
Male	660.0 (290.0–1684.0)	0.38 (0.29–0.54)	764.0 (352.0–1868.5)	0.42 (0.33–0.56)
Female	702.0 (284.0–2000.0)	0.42 (0.33–0.53)	1118.0 (295.0–3080.0)	0.48 (0.35–0.59)
*P*	0.769	0.234	0.507	0.558
Age (range), years				
≤60	702.0 (260.0–1868.5)	0.39 (0.31–0.53)	802.0 (287.0–2430.0)	0.47 (0.33–0.56)
>60	656.0 (355.0–1847.0)	0.41 (0.30–0.55)	888.0 (384.0–2220.0)	0.43 (0.34–0.58)
*P*	0.414	0.856	0.668	0.965
Stage				
I–IIA	482.0 (238.5–1081.0)	0.34 (0.28–0.47)	488.0 (262.8–1048.0)	0.35 (0.29–0.48)
IIB–IV	896.0 (351.0–2330.0)	0.43 (0.34–0.56)	1395.0 (449.5–2720.0)	0.52 (0.41–0.62)
*P*	*0.003*	*0.0001*	*0.006*	*<0.0001*
B-symptoms				
No	496.0 (267.0–1426.0)	0.39 (0.29–0.52)	523.0 (293.0–1672.8)	0.41 (0.31–0.54)
Yes	736.0 (348.0–2240.0)	0.41 (0.32–0.54)	1657.0 (433.0–3520.0)	0.52 (0.39–0.63)
*P*	0.104	0.251	*0.034*	*0.013*
Bulky disease				
No	662.0 (287.0–1661.5)	0.39 (0.30–0.53)	783.0 (320.5–2330.0)	0.43 (0.34–0.55)
Yes	1011.0 (269.0–2434.5)	0.42 (0.31–0.59)	1148.0 (334.5–2161.8)	0.45 (0.35–0.61)
*P*	0.431	0.406	0.741	0.737
Lactate dehydrogenase				
Normal	472.0 (230.8–978.0)	0.37 (0.29–0.49)	455.0 (248.5–1114.0)	0.39 (0.30–0.52)
Elevated	1200.0 (400.0–3020.0)	0.43 (0.33–0.59)	1629.5 (517.0–3520.0)	0.52 (0.38–0.63)
*P*	*<0.0001*	*0.006*	*<0.0001*	*0.003*
Prognostic score				
Low risk	482.0 (248.0–1024.0)	0.37 (0.29–0.51)	522.0 (267.5–1771.0)	0.41 (0.30–0.52)
High risk	942.0 (402.0–2600.0)	0.42 (0.34–0.57)	1620.0 (493.0–2870.0)	0.52 (0.39–0.66)
*P*	*0.001*	*0.01*	*0.002*	*0.005*

Data were presented as median (IQR 25–75); IQR, interquartile range; circulating cell-free DNA analysis included in concentration and DII levels; DII, DNA integrity index. The IPI score was used for patients with non-Hodgkin’s lymphoma and the IPS score for patients with Hodgkin’s lymphoma. IPI scores 0 and 1 were considered low risk, 2 and 3 as high risk, while with IPS scores 0–2 were considered as low risk and 3 as high risk

*P* value, comparison between the two strata of each individual variable. *P* values below 0.05 were shown in italics

### Prognostic significance of circulating cell-free DNA in diffuse large B cell lymphoma patients

**Fig. 3:**
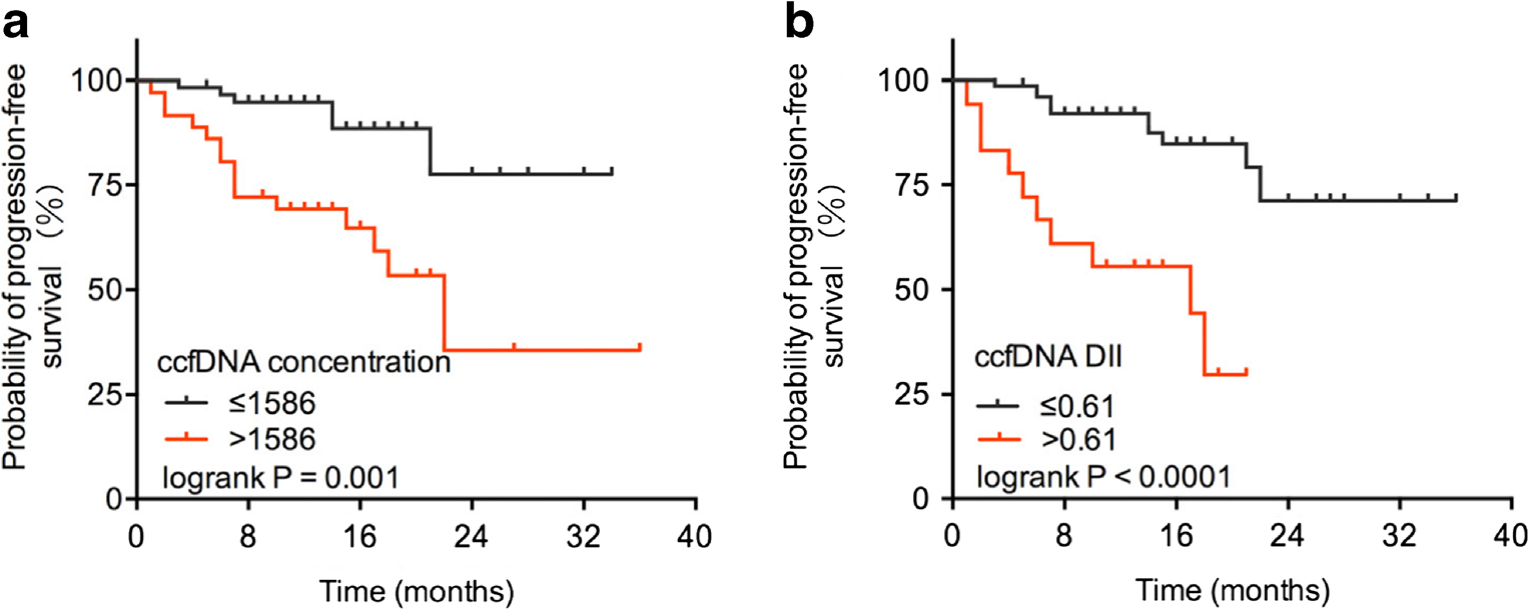
Survival curves of DLBCL patients stratified by circulating cell-free DNA levels. Kaplan-Meier survival curve and log-rank test according to **a** concentration and **b** DII determined by ccfDNA analysis were using the cut-offs identified by means of ROC for 2-year progression-free survival (*P* value noted in the figure). ccfDNA, circulating cell-free DNA; DII, DNA integrity index; ROC, receiver operating characteristic

The role of the ccfDNA levels at the time of diagnosis as a prognostic marker was analyzed in patients with DLBCL, who constituted the largest diagnostic entity included in this study. The most discriminatory cut-offs for 2-year PFS were identified by ROC. The ccfDNA concentration and the DII were analyzed as dichotomic variables using 1586 ng/ml and 0.61 as the cut-off points, respectively. As shown in the Kaplan-Meier curves for the different levels of ccfDNA concentration, patients with a ccfDNA concentration >1586 ng/ml had a 2-year probability of PFS of only 44% (95% CI 15–73%), whereas patients with a ccfDNA concentration ≤1586 ng/ml had significantly higher probability of 2-year PFS at 78% (95% CI 55–99%; *P* = 0.001; Fig. 3a). The prognostic value of ccfDNA fragmentation on PFS was also evaluated using the DII. Patients showing a DII >0.61 had a 59% probability of 2-year PFS (95% CI 38–79%), which was significantly shorter than that in patients with a DII ≤0.61 (87%, 95% CI 69–100%, *P* < 0.0001; Fig. 3b).

Following the univariate analysis, we found that both the ccfDNA concentration and the DII were associated with PFS; moreover, advanced stage, B-symptoms, and elevated LDH levels were significant adverse factors (Table 3). Thus, these variables were assessed in a multivariate Cox regression model, which showed that the DII appeared to be a statistically independent prognostic factor (HR = 3.04, 95% CI 1.197–7.696; *P* = 0.019), whereas the ccfDNA concentration was not significant in this multivariate analysis (HR = 1.45, 95% CI 0.490–4.263; *P* = 0.504).

**Table 3:** Univariable Cox regression models for progression-free survival (PFS) in diffuse large B cell lymphoma patients

	HR (95% CI)	*P* value
Gender (male)	1.128 (0.454–2.799)	0.796
Age (>60 years)	1.196 (0.495–2.886)	0.692
Advanced stage	7.977 (1.856–34.29)	*0.005*
B-symptoms	6.639 (2.404–18.33)	*<0.0001*
Bulky disease	0.571 (0.133–2.456)	0.452
Elevated LDH	4.545 (1.522–13.57)	*0.007*
Elevated ccfDNA		
Concentration	4.270 (1.653–11.03)	*0.003*
DII	5.165 (2.187–12.19)	*<0.0001*

Data were presented as HR (95% CI); HR, hazard ratio; CI, confidence interval. ccfDNA, circulating cell-free DNA; elevated ccfDNA was included in both concentration and DII levels

*P* values below 0.05 were shown in italics

## Discussion

This study reveals that compared to healthy individuals, patients with lymphoma frequently have higher concentrations and longer strands of ccfDNA at the time of diagnosis, which correlates with clinical parameters and was demonstrated to be a negative predictor of DLBCL patients’ outcome.

Recently, studies conducted in various malignancies have shown that ccfDNA levels are significantly increased in cancer [22, 23]. Our work revealed that the ccfDNA concentration and the DII levels were elevated in patients with lymphoma. The analysis of the histological subsets revealed that the ccfDNA concentration and the DII levels varied according to the lymphoma subtype. Unfortunately, the B-NHL and T-NHL cohorts were insufficient to perform a significant histological subtype analysis.

There are only few reports that studied the clinical impact of ccfDNA levels on lymphoma patients and were mainly oriented to analyze the correlation between an increase in the ccfDNA concentration with disease risk and adverse clinical performance. To the best of our knowledge, other analytical parameters exhibited by ccfDNA such as the DII have not been studied in lymphoma patients. Here, we confirmed the usefulness of detecting the quantity and quality of ccfDNA as a diagnostic tool for lymphoma. Data from our ROC analysis showed that the AUC of the concentration to distinguish HL, DLBCL, and NK/TCL patients from normal controls was 0.75, 0.86, and 0.79, respectively; furthermore, the AUC of the DII was higher than that of the concentration (0.83, 0.90, and 0.81 for HL, DLBCL, and NK/TCL, respectively), indicating that the ccfDNA concentration alone appeared to be insufficient for a high-quality diagnostic performance. Hohaus et al. [17] conducted a diagnostic analysis of the ccfDNA concentration for patients with HL and DLBCL and found that the maximum sensitivity and specificity did not exceed 75%, making this parameter an unlikely candidate for lymphoma screening. Our study found that for DLBCL patients, there was an added incremental diagnostic value when the ccfDNA concentration was combined with the DII.

Regarding the clinical correlation of ccfDNA levels in lymphoma patients, we observed an association between the ccfDNA levels and a number of clinical parameters that indicate a worse prognosis such as advanced stage disease, the presence of B-symptoms, elevated LDH levels, and high IPI score; these correlations suggest that both the ccfDNA concentration and the DII might reflect actively proliferating disease and lymphoma burden. Nevertheless, a literature analysis highlights the confusion regarding these ccfDNA parameters with tumor burden and predicting a therapeutic response. Hohaus et al. [17] also observed significant associations between the ccfDNA concentration and some adverse parameters, whereas a study by Jones et al. [18] found that the ccfDNA concentration was not indicative of lymphoma burden once therapy had commenced; they reported that only lymphoma-specific DNA such as Epstein-Barr virus DNA could be used to monitor the disease response in lymphoma patients. In addition, both the ccfDNA concentration and the DII were not significantly different between germinal center B cell type and non-germinal center B cell type DLBCL based on the Hans classification system (data not shown).

We assessed the prognostic significance of ccfDNA levels in patients with DLBCL. Our data showed that high concentrations of ccfDNA and an elevated DII were strongly correlated with poor outcome in patients with DLBCL, which has already been reported [17]. However, our study data revealed that the ccfDNA concentration was insufficient as an independent prognostic factor compared to other existing and validated adverse factors such as disease stage or elevated LDH levels [24].

The origin of ccfDNA is still unclear. In healthy individuals, the ccfDNA concentration is low, which is ascribed to the efficient removal of most nonliving cells from circulation by phagocytes. Schwarzenbach et al. [25] proposed that ccfDNA could be released by either apoptosis or necrosis and found that the predominant fragment length of ccfDNA frequently occurred in multiples of 180 bp, which is typical of DNA released from apoptotic cells. However, the presence of larger fragments such as those found in cancer patients suggested that ccfDNA could also be derived from necrotic cells. Moreover, some studies have attributed a notable fraction of ccfDNA to active release from lymphocytes [26]. ccfDNA is not specific to neoplastic conditions as increased levels have also been identified in inflammatory and autoimmune diseases. An association between a high concentration of ccfDNA and poor prognosis may be linked to tumor burden [27]; however, changes in the total ccfDNA concentration may reflect not only changes in circulating tumor DNA (ctDNA) but also in other conditions that may lead to an increase in ccfDNA, including infection or chemotherapy drug-induced release of normal ccfDNA into the circulation. ctDNA is a specific ccfDNA released by tumor cells, which contains genetic and epigenetic alterations concordant with those of the primary tumor. This may explain why our data showed the levels of ccfDNA in HL patients were as high as in NHL patients and indicated that the overall concentration of ccfDNA was not an independent significant prognostic factor of survival to some extent. Actually, our results showed the limitations of analyzing only the total ccfDNA levels, as focusing on tumor-specific ccfDNA might serve as a more adequate surrogate biomarker. For solid tumors, many studies have validated that genetic alterations detected in ctDNA correspond to the primary tumor and that the levels of ctDNA could assess the tumor response and even provide an earlier indication of disease progression [28–30]. Roschewski et al. [31] reported that monitoring ctDNA could identify patients with DLBCL who are at risk of recurrence before the manifestation of clinical evidence of disease, and interim ctDNA may be a promising biomarker to identify patients at high risk of treatment failure. However, additional studies on ctDNA are required to prove its value in clinical practice. Thus, as the ccfDNA levels could still reflect disease progression [32], they remain a useful biomarker to monitor disease status, especially when combined with other parameters such as the DII or ctDNA levels.

In this study, it should be noted that ccfDNA extraction was performed using the magnetic bead method, which is based on the principle of nucleic acid adsorption and release via magnetic beads. The most commonly used DNA extraction methods include the phenol-chloroform method, sodium iodide method, and magnetic bead method as well as the use of commercial DNA isolation kits [33]. Pan et al. [34] compared the phenol-chloroform method, commercial kits, and magnetic bead method in the detection of mycobacterium tuberculosis DNA and found that the magnetic bead method had the highest DNA extraction efficiency and the best reproducibility.

In conclusion, our study demonstrated that the quantification and integrity analysis of ccfDNA from the plasma of patients with lymphoma might be a useful noninvasive technique for clinical practice. In patients with DLBCL, high levels of ccfDNA and an elevated integrity index were associated with poor prognosis, although, based on our results, only the DII was an independent adverse factor for PFS. These results suggest that additional prospective studies with larger cohorts and a longer follow-up should be initiated to assess the utility of ccfDNA analysis, especially using tumor-specific ccfDNA.
